# Effects of Alternative Blood Sources on *Wolbachia* Infected *Aedes aegypti* Females within and across Generations

**DOI:** 10.3390/insects9040140

**Published:** 2018-10-11

**Authors:** Véronique Paris, Ellen Cottingham, Perran A. Ross, Jason K. Axford, Ary A. Hoffmann

**Affiliations:** Pest and Environmental Adaptation Research Group, School of BioSciences, Bio21 Institute, University of Melbourne, Melbourne, VIC 3010, Australia; Veronique_paris@hotmail.de (V.P.); ellenpc@student.unimelb.edu.au (E.C.); perran.ross@unimelb.edu.au (P.A.R.); jkaxford@unimelb.edu.au (J.K.A.)

**Keywords:** mosquitoes, *Aedes aegypti*, *Wolbachia*, blood meal

## Abstract

*Wolbachia* bacteria have been identified as a tool for reducing the transmission of arboviruses transmitted by *Aedes aegypti*. Research groups around the world are now mass rearing *Wolbachia*-infected *Ae. aegypti* for deliberate release. We investigated the fitness impact of a crucial element of mass rearing: the blood meal required by female *Ae. aegypti* to lay eggs. Although *Ae. aegypti* almost exclusively feed on human blood, it is often difficult to use human blood in disease-endemic settings. When females were fed on sheep or pig blood rather than human blood, egg hatch rates decreased in all three lines tested (uninfected, or infected by *w*Mel, or *w*AlbB *Wolbachia*). This finding was particularly pronounced when fed on sheep blood, although fecundity was not affected. Some of these effects persisted after an additional generation on human blood. Attempts to keep populations on sheep and pig blood sources only partly succeeded, suggesting that strong adaptation is required to develop a stably infected line on an alternative blood source. There was a decrease in *Wolbachia* density when *Ae. aegypti* were fed on non-human blood sources. Density increased in lines kept for multiple generations on the alternate sources but was still reduced relative to lines kept on human blood. These findings suggest that sheep and pig blood will entail a cost when used for maintaining *Wolbachia*-infected *Ae. aegypti*. These costs should be taken into account when planning mass release programs.

## 1. Introduction

The majority of female mosquito species require a vertebrate blood meal to obtain nutrients for successful egg development [1], with some species being explicitly adapted to humans [2,3]. Mosquito species such as *Aedes aegypti* are highly anthropophilic and are the key species involved in transmission of human pathogens such as dengue [4]. *Ae. aegypti* inhabits predominantly urban environments which currently places approximately half of the world’s population at risk of infection from dengue alone [5,6].

An emerging approach for control of dengue and other arbovirus diseases involves the release of mosquitoes infected with the endosymbiotic bacterium *Wolbachia* to reduce the transmission of the virus within human communities [7,8]. The *Wolbachia* bacterium is a maternally inherited endosymbiont that reduces the transmission of arboviruses in the *Ae. aegypti* host [9,10,11]. The exact mechanism that allows *Wolbachia* to induce this phenomenon is still unclear. However, factors such as a rise in host immune response, elevation in host methyltransferase to reduce viral production and suppression of key lipids required for viral replication have been identified as potentially important [12,13,14].

*Wolbachia* strains from other arthropod hosts have been successfully transinfected into *Ae. aegypti*, causing a range of phenotypic outcomes [15,16,17,18]. The *Wolbachia* infections *w*Mel and *w*AlbB induce cytoplasmic incompatibility (CI) in *Ae. aegypti*, resulting in embryo death when *Wolbachia*-infected males mate with uninfected females [16]. This provides a significant reproductive advantage for *Wolbachia*-infected compared to uninfected female mosquitoes. Maternal transmission and cytoplasmic incompatibility combined allow *Wolbachia* infected mosquitoes to invade an uninfected population [19].

*Wolbachia* programs are initiated to suppress dengue transmission through replacing uninfected populations by infected ones or through suppressing mosquito populations using CI. Both strategies require a large number of laboratory-reared mosquitoes with a high level of fitness under field conditions to be successful. Blood feeding is an integral part of mosquito colony maintenance. *Ae. aegypti* is highly adapted to human blood; however, in many countries, large volumes of human blood are difficult to obtain and subject to strict ethical and regulatory control [20], particularly given the risk of viral transfer in disease-endemic zones. Artificial blood sources are currently under development [21] and appear to act as appropriate substitutes for human blood [22] including for *w*Mel-infected mosquitoes where no effect on *Wolbachia* parameters including density, CI and virus inhibition were demonstrated [20,23]. However, issues relating to insufficient storage time [20] or reduced egg hatch rates [23] are reasons why alternative blood sources continue to be used in different locations. Where these are used, they may affect the success of releases by (1) directly decreasing the fitness of released mosquitoes, if feeding produces sub-optimal offspring that cannot compete effectively with wild mosquitoes, (2) promoting a process of genetic adaptation to non-human blood that has negative fitness consequences for released mosquitoes when feeding on human blood again, and (3) reducing *Wolbachia*-related effects like maternal transmission, CI and viral blockage [24].

Here, we aimed to assess the use of non-human blood meals on *Ae. aegypti*. Previous research has shown that non-human blood sources influence the fitness of *Wolbachia*-infected mosquitoes. For instance, *w*MelPop-infected *Ae. aegypti* fed on mouse, guinea pig and chicken blood results in a sharp decrease in fecundity and egg hatch rates, in contrast to uninfected mosquitoes [25]. This raises the issue of whether there will be significant negative effects of various non-human blood sources used around the world for *Ae. aegypti* cultures.

We explore the following questions: What is the immediate impact of two common blood sources (sheep and pig) on different *Wolbachia* infection types, given that *w*Mel and *w*AlbB are now being successfully used in releases? Do the negative effects of one generation of feeding persist into the next generation if mosquitoes subsequently feed on human blood? Can large outbred populations adapt to different blood sources, and if so does this mean that performance on human blood is negatively affected following adaptation? We characterised key fitness characteristics including fecundity, egg hatch rate and larval development parameters. We also assessed *Wolbachia* density to investigate if *Ae. aegypti* fed on non-human blood sources experienced a loss or reduction of the *Wolbachia* infection.

## 2. Materials and Methods

### 2.1. Wolbachia Strains and Laboratory Conditions

We used *Ae. aegypti* that were uninfected or infected with the *Wolbachia* strains *w*Mel and *w*AlbB in our experiments. The *w*Mel and *w*AlbB strains are currently being reared for mass release and are described in Walker et al. [16], Xi et al. [17] and Axford et al. [26]. Prior to experimentation, *Wolbachia*-infected females were crossed to uninfected Australian field caught *Ae. aegypti* males for three generations to control for genetic background effects. Each colony was maintained in a temperature-controlled insectary at 26 °C ± 1 °C with a 12 h photoperiod as described by Axford et al. [26] and Ross et al. [27]. Colonies were provided with constant access to a 10% sucrose solution and maintained in 15 cm^3^ cages (Bugdorm model #41515 Insect Rearing Cages©, Megaview Science C., Ltd., Taichung, Taiwan) and enclosed in plastic bags to maintain high humidity. Adult females were fed on a human volunteer’s arm once per generation to initiate egg laying. Colonies were maintained in two replicate cages containing approximately 100 mosquitoes each.

### 2.2. Experimental Design

The study was comprised of two components. In the first component, the direct and intergeneration effects of blood feeding were examined. The *w*Mel, *w*AlbB and uninfected populations were randomly allocated at the larval stage into three treatments, each with 200 larvae raised with abundant food (approximately 0.5 mg/larva/day [27]). Pupae were sexed to ensure equal sex ratios. Experiments with different combinations of blood source and *Wolbachia* infection type were initially conducted in groups of 25 females and 25 males. Seven-day-old females were provided with a human, pig or sheep blood meal. Mosquitoes fed on each blood source were then scored for fecundity, egg hatch rate and *Wolbachia* density. To test for persistent effects of blood feeding, F1 offspring from females fed on different blood sources were then fed on human blood and re-tested for fecundity, egg hatch rate and *Wolbachia* density.

In the second component, we determined if adaptation to non-human blood sources was possible and examined the costs associated with this process. *Aedes aegypti* colonies that were uninfected or carrying the *w*AlbB or *w*Mel infections were maintained on pig, sheep or human blood for four generations. We attempted to maintain a large population size in these experiments to avoid deleterious inbreeding effects that can be substantial in *Ae. aegypti* by turning over 200+ adults each generation, but terminated lines once these fell below 20 because inbreeding effects then make line comparisons difficult [28]. This experiment was run on unreplicated lines because of resource limitations. Unfortunately, some lines were lost in this experiment presumably due to the negative effects of maintaining mosquitoes on alternate blood sources (see below) which may have been cumulative across time. The *w*Mel and *w*AlbB lines maintained on sheep were lost at the second and third generation, respectively. The *w*Mel line maintained on pig blood was lost at the F4 stage when <20 larvae developed. The remaining lines were scored for egg hatch rate and development time after four generations (F4). *Wolbachia* density was also scored at the start of this experiment in F1 offspring after parents had been held for a generation on different blood sources (i.e., before any lines were lost).

We continued the surviving lines on their respective blood sources for an additional six generations (i.e., 10 generations in total), with no further lines lost because large population sizes had developed by this stage and these were easily maintained. To test for any deleterious effects of the selection process, mosquitoes from these lines were fed on human blood and retested for fecundity, egg hatch rate and *Wolbachia* density.

### 2.3. Blood Feeding

Pig and sheep blood were considered for their potential as an alternative blood source to human blood because of their ready availability in some countries where releases are planned or being undertaken. Fresh sheep and pig blood were collected from JBS Australia© (Brooklyn, NSW, Australia) and Diamond Valley Pork© (Melbourne, VIC, Australia), respectively, into 10 mL Lithium Heparin Vacuette evacuated blood collection tubes (Greiner Bio-One International, Kremsmünster, Austria) and stored at 4 °C. Before blood collection, animals were kept for two weeks without access to antibiotic-containing food and water sources to ensure that the blood was mostly free of antibiotics. Non-human blood sources were used in experiments within the day of collection or the day after. Human blood was sourced from the Red Cross (Agreement #16-10VIC-02) and refreshed monthly. Since we used different batches of blood across experiments, we cannot rule out the influence of batch effects on the results. Colonies were tested after being maintained on their respective blood sources or being transferred back to human blood. The *w*Mel groups fed on sheep and pig blood and the *w*AlbB group fed on sheep blood were not tested later because population sizes declined, resulting in insufficient egg numbers to allow the populations to persist without severe inbreeding effects being anticipated [28].

Mosquitoes were fed via Hemotek© membrane feeders (Discovery Workshops, Accrington, UK) [29]. To prepare the blood-filled Hemotek© disc, a 6 cm × 6 cm square of collagen membrane (Discovery Worskshops, Accrington, UK) was cut out and placed over the metal reservoir. The membrane was then fastened with an O ring. Approximately 6 mL of blood was pipetted into the metal reservoir that was plugged with two nylon stoppers. The blood-filled reservoir was screwed onto the heat-supplying feeder, which was calibrated to 37 °C (human body temperature). The completed feeder was placed on top of the mosquito colony cage. Colonies were given approximately 20–25 min to feed, or until all females showing an interest in feeding by probing the blood delivery site with their proboscis had taken a blood meal (indicated by an engorged abdomen). Blood meal reservoirs were restricted to *Wolbachia* infection types; there was no sharing of blood reservoirs between infection types to avoid the unlikely event of cross-contamination.

### 2.4. Fecundity and Egg Hatch Rate

Fecundity was measured by isolating engorged females. For the first component looking at parental effects following feeding on different blood sources or subsequent feeding on human blood, five to six females per replicate (4 cages per *Wolbachi*a infection type/blood source group) were taken (for a total of 20–24 females for each *Wolbachia* infection/blood source group). For the second component (following selection on different blood sources), 2–3 females per replicate were taken in the F4 generation from each of three cages (for a total of 5–9 females per *Wolbachia* infection/blood source group). More females were available at the F10 generation for testing when 16–24 females were used per blood source.

For the assays, engorged females were isolated in 70 mL cups filled halfway with reverse osmosis (RO) water and lined with sandpaper (Norton^®^ Master Painters P80, Saint-Gobain Abrasives Pty. Ltd., Thomastown, Victoria, Australia) as an oviposition substrate. The top of the cup was covered with mesh to prevent mosquitoes escaping. Females were allowed one week to lay eggs before being stored in 70% ethanol and sandpaper strips were collected and partially dried. Three days after collection, eggs were hatched in plastic trays filled with 500 mL of RO water. Two days later, fecundity and egg hatch rates were calculated by counting the number of hatched and unhatched eggs. An egg was recorded as ‘hatched’ if the egg cap was detached.

### 2.5. Development Time

After lines had been cultured for several generations on different blood sources as part of the second component, we noticed that there was a delay in larval development. We quantified development time at the F4 generation as the duration taken from hatching to adult emergence. Larvae were selected at random using a pipette and placed into trays filled with 150 mL of RO water where they were given access to TetraMin^®^ fish food (Tetra, Melle, Germany) ad libitum (approximately 0.5 mg/larvae/day). Trays of larvae were maintained in incubators at 26 °C ± 1 °C. Trays were rotated daily to account for any variation in temperature or light within the incubators. Trays were checked twice daily where the number and sex of emerging adults was recorded. Development time was scored on 4–22 individuals of each sex per line from a particular blood source.

### 2.6. DNA Extraction and Wolbachia Screening

We extracted genomic DNA using Chelex^®^ 100 Resin (Bio-Rad Laboratories, Hercules, CA, USA). Adults were removed from cages for extraction one week after blood feeding to ensure that eggs had been laid. Extraction was conducted using 250 µL of 5% Chelex^®^ and 3 µL of Proteinase K (20 mg/mL) (Roche Diagnostics Australia Pty. Ltd., Castle Hill, NSW, Australia) solution. Individual samples were ground using two 3 mm glass beads per sample in a mixer mill (Retsch^®^ GmbH, Haan, Germany). Samples were then incubated for 60 min at 65 °C and 10 min at 90 °C. Samples were diluted by 1:10 then stored at −20 °C.

We used a LightCycler^®^ 480 (Roche Applied Science, Indianapolis, IN, USA) to confirm *Wolbachia* infection status and estimate *Wolbachia* density. Three primer sets were used to amplify markers specific to mosquitoes, differentiate *Ae. aegypti* from other *Aedes* species and amplify markers specific to the *Wolbachia* infections (*w*Mel or *w*AlbB) as described by Lee et al. [30] and Axford et al. [26], where forward and reverse primer sequences can be found. Comparisons between these three sets of markers were based on crossing point (Cp) values and temperature melt curve (Tm) analysis. Three technical replicates were completed for each mosquito; differences in crossing point between the *Ae. aegypti*-specific and *Wolbachia*-specific markers were averaged to obtain an estimate of *Wolbachia* density (once adjusted for the *Aedes* control gene Cp value). A detailed qPCR protocol is provided in Yeap et al. [31] and Lee et al. [30].

We estimated the density of the *w*Mel and *w*AlbB infections from each generation and blood source by comparing *Ae. aegypti* and *Wolbachia* Cp values. The *Wolbachia* Cp value (Cp2) was subtracted from the *Ae. aegypti* Cp value (Cp1) as a relative estimate of *Wolbachia* density. If we assume that the efficiency of the reaction is perfect (i.e., R = 2^−ΔΔCp^), the difference in Cp values between samples can give an estimate of the relative concentration of *Wolbachia* DNA relative to mosquito DNA when computed as 2^(Cp1−Cp2)^. While Cp values do not give definitive quantification, they provide a relative guide as to the concentration of a *Wolbachia* infection when compared to the host. However, in other work, we have found a good agreement between relative *Wolbachia* densities established through Cp values with those obtained via a Droplet Digital system that directly measures absolute copy number [32].

### 2.7. Statistical Analysis

Statistical analysis was completed using SPSS V21.0 for Windows (SPSS Inc., Chicago, IL, USA) and R (3.3.3) [33]. All data sets were tested for normality using Shapiro–Wilks tests. Data sets were analysed through general linear models with and without transformation, although in no case did the transformation influence the conclusions from the analysis. For the results, analyses on egg hatch rates are presented after these had been angular transformed, while analyses of fecundity data are presented after these were ln(x + 1) transformed; however, both variables are displayed as box plots on the untransformed data. Tukey’s b post hoc test was undertaken for pairwise comparison between blood sources and *Wolbachia* infection type where appropriate. Density data (*Wolbachia* density relative to *Aedes* controls) were also analysed through general linear models.

## 3. Results

### 3.1. Immediate and F1 Effects (Component 1)

#### 3.1.1. Fitness Effects

**Figure 1:**
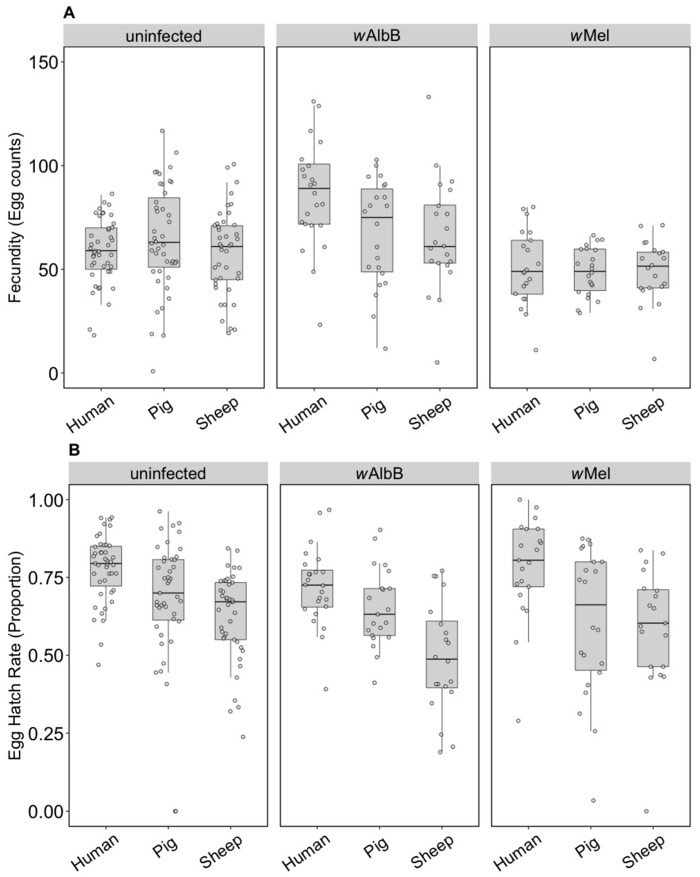
Comparison of fecundity and egg hatch rates across infection types (uninfected, *w*Mel, *w*AlbB) and blood sources (human, pig, sheep). (**A**) Fecundity (egg counts); (**B**) egg hatch rate (proportion). Each dot represents the egg count and egg hatch rate of an individual female. The boxplots represent the first to the third quartiles around the median (horizontal grey line) and the vertical bars represent the 1.5 interquartile of the lower and upper quartiles. Around 24 females per blood source and generation were tested.

The fecundity of the three infection types (uninfected, *w*Mel and *w*AlbB) was not affected by the blood source (General Linear Model: F_(2, 279)_ = 2.13, *p* = 0.121). While there was no interaction between *Wolbachia* infection type and blood source (F_(4, 279)_ = 0.84, *p* = 0.503), there was an effect of the infection type overall (F_(2, 279)_ = 3.27, *p* = 0.040) reflecting the fact that the *w*AlbB strain had somewhat higher fecundity than *w*Mel in this experiment (Figure 1A).

In contrast to fecundity, we found a significant effect of the blood source (General Linear Model: F_(2, 246)_ = 29.32, *p* < 0.001) on egg hatch rates. All *Wolbachia* infection types (uninfected, *w*Mel and *w*AlbB) fed on non-human blood sources showed a significant decrease in egg hatch rate compared to mosquitoes fed on human blood (post hoc tests, all *p* < 0.001) (Figure 1B). There was also an effect of *Wolbachia* infection type (F_(2, 246)_ = 4.79, *p* = 0.009) but no interaction between infection type and blood source (F_(4, 246)_ = 0.19, *p* = 0.941). Post hoc tests indicated that *w*AlbB egg hatch rate was lower than that of the uninfected group, while *w*Mel overlapped with both (Figure 1B). The total fertility of a female fed on sheep blood as measured by the number of hatched eggs was 36.3% lower on average than a female fed on human blood, while pig blood feeding reduced fertility by 16.5%.

**Figure 2:**
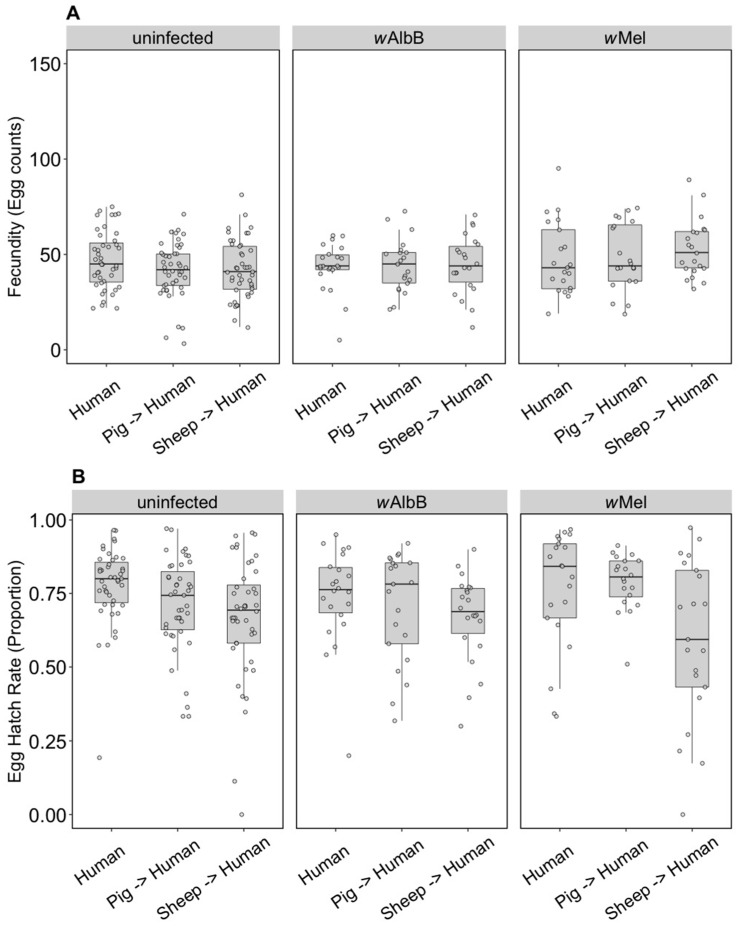
Comparison of fecundity and egg hatch rates across infection types (uninfected, *w*Mel, *w*AlbB) when females were fed on human blood but parents were fed on different blood sources (human, pig, sheep). (**A**) Fecundity (egg counts); (**B**) egg hatch rate (proportion). Each dot indicates the egg count and egg hatch rate of an individual female. The boxplots represent the first to the third quartiles around the median (horizontal grey line) and the vertical bars represent the 1.5 interquartile of the lower and upper quartiles. Around 24 females per blood source and generation were tested.

The F1 generation of parents fed on non-human blood sources showed persistent effects of parental blood feeding despite F1 exposure to human blood. There was no effect of parental blood source (F_(2, 279)_ = 0.40, *p* = 0.671), infection type (F_(2, 279)_ = 0.03, *p* = 0.968) or an interaction effect (F_(4, 279)_ = 0.083, *p* = 0.99) on (ln (x + 1)) fecundity (Figure 2A). However, (angular transformed) egg hatch rates were affected by parental blood source (F_(2, 247)_ = 5.48, *p* = 0.005), with reduced egg hatch rates when parents were fed on pig or sheep blood (Figure 2B). There were no effects of the infection type (F_(2, 247)_ = 0.24, *p* = 0.789) and no interaction between infection type and blood source (F_(4, 247)_ = 1.06, *p* = 0.375) on egg hatch rates. The fertility of a female whose mother had fed on sheep blood as measured by the number of hatched eggs was 16.8% lower on average than for females fed on human blood in both generations, while pig blood feeding by a mother reduced the fertility of the daughter by 7.9%.

#### 3.1.2. *Wolbachia* Density

**Figure 3:**
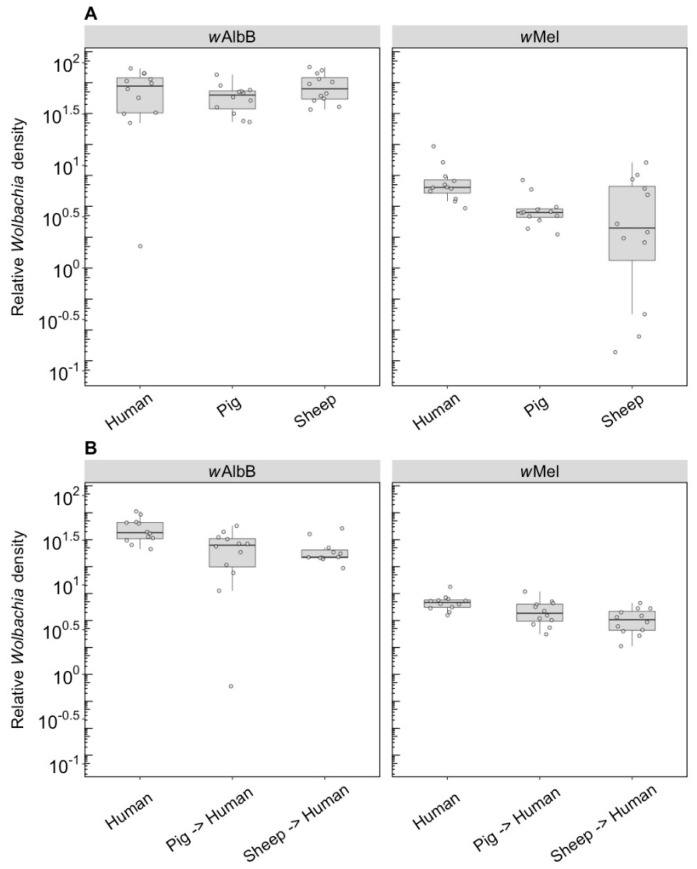
Comparison of the relative *Wolbachia* density of the infections types (*w*Mel, *w*AlbB) across different blood sources (human, pig, sheep). (**A**) Relative *Wolbachia* density of parents fed on different blood sources; (**B**) relative *Wolbachia* density of F1s after being re-introduced to human blood. Each dot represents the density of an individual mosquito. The boxplots represent the first to the third quartiles around the median (horizontal grey line) and the vertical bars represent the 1.5 interquartile of the lower and upper quartiles. Around 12 individuals per blood source and infection type were tested from the parental and F1 generations.

We compared the *Wolbachia* density of parents fed on different blood sources and the density of F1s fed on human blood. For *w*AlbB in the parents, there was no effect of blood source on *Wolbachia* density (F_(2, 33)_ = 1.14, *p* = 0.333), but there was for *w*Mel (F_(2, 33)_ = 5.08, *p* = 0.012), with density being lower when females were fed on pig and sheep blood (Figure 3A). The *w*AlbB density when F1s were fed on human blood was affected by parental blood source (F_(2, 33)_ = 9.22, *p* = 0.001); density was lower when parents were fed on both pig and sheep blood (Figure 3B), with both treatments differing in post hoc tests from the human blood treatment. There was also a difference in density between the F1s for *w*Mel (F_(2, 33)_ = 8.14, *p* = 0.001), with a reduced density when parents were fed on pig and sheep blood even though F1s had been fed on human blood (Figure 3B).

### 3.2. Long-Term Effects (Component 2)

#### 3.2.1. Fitness Effects (F4)

**Figure 4:**
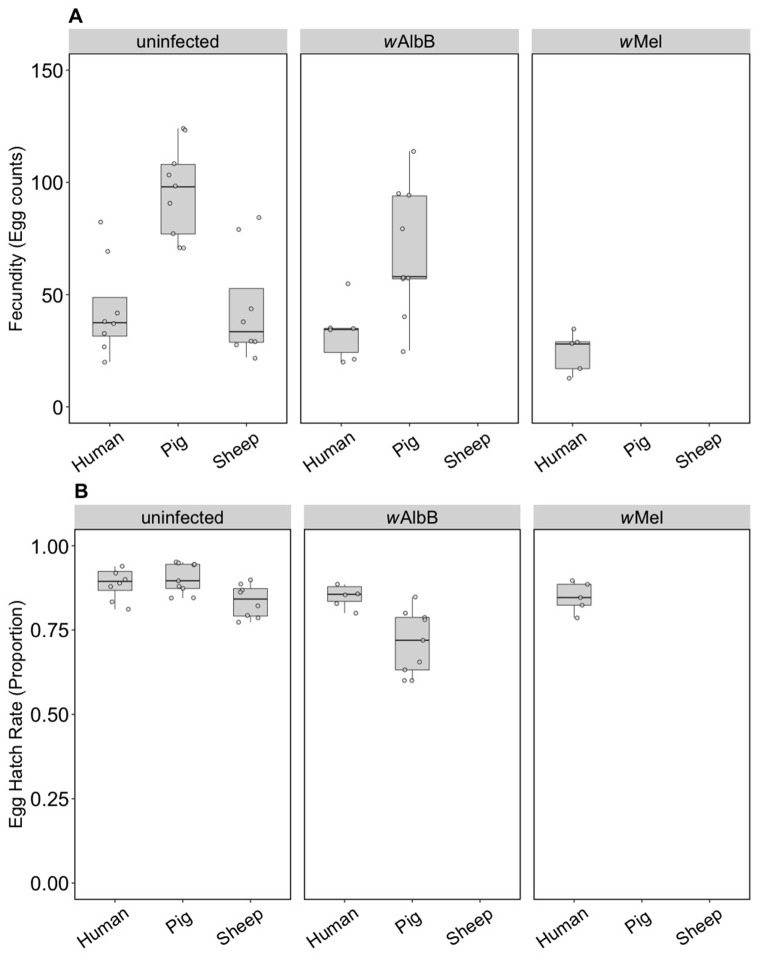
Comparison of fecundity and egg hatch rates across infection types (uninfected, *w*Mel, *w*AlbB) and different blood sources (human, pig, sheep) of F4 selected groups. (**A**) Fecundity (egg counts); (**B**) egg hatch rate (proportion). Each dot embodies the egg count and egg hatch rate of an individual female with 5–9 females tested per treatment. The boxplots represent the first to the third quartiles around the median (horizontal grey line) and the vertical bars represent the 1.5 interquartile of the lower and upper quartiles.

We compared the fecundity and egg hatch rates of lines kept on the three blood sources for four generations. Note that the number of females in these tests (5–9) was lower per treatment group than in the earlier trials, which was a reflection of the difficulty we had in maintaining mosquitoes on the different blood sources. All infection types performed well on human blood and there were no differences in fecundity (F_(2, 16)_ = 2.55, *p* = 0.110) (Figure 4A) or egg hatch rate (F_(2, 16)_ = 0.757, *p* = 0.485) (Figure 4B) between the infection types. For groups maintained on pig blood, both fecundity (F_(1, 16)_ = 5.41, *p* = 0.033) and egg hatch rate (F_(1, 16)_ = 32.65, *p* < 0.001) were lower in *w*AlbB mosquitoes compared to uninfected mosquitoes (Figure 4A,B).

For the uninfected group maintained on pig or sheep blood, fecundity differed between blood sources (F_(2, 22)_ = 13.14, *p* < 0.001) and was higher when mosquitoes were fed on pig blood compared to human or sheep (Figure 4A), though egg hatch rates did not differ significantly (F_(2, 22)_ = 3.21, *p* = 0.060) (Figure 4B). This resulted in a 127% increase in viable eggs when mosquitoes fed on pig versus human blood and a small reduction of 4.5% for sheep. Likewise, *w*AlbB exhibited significantly higher fecundity on pig compared to human blood (F_(1, 13)_ = 8.87, *p* = 0.011), but egg hatch rates were lower (F_(1, 13)_ = 10.05, *p* = 0.007); however, there was still a 69.9% increase in larval number on pig blood. These results contrast to those obtained in the parental generation, suggesting that there is blood source adaptation in the lines.

**Figure 5:**
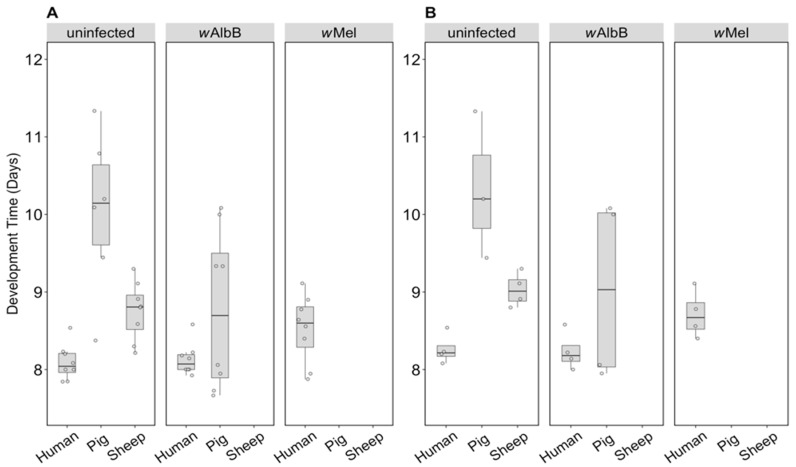
Comparison of development time across infection types (uninfected, *w*Mel, *w*AlbB) and different blood sources (human, pig, sheep). The development time in days, calculated as the number of days from being hatched (day 0) to emerging as adults was measured for (**A**) males and (**B**) females. Individual data points indicate the average development time of mosquitoes in each replicate based on 4–22 individuals. The boxplots represent the first to the third quartiles around the median (horizontal grey line) and the vertical bars represent the 1.5 interquartile of the lower and upper quartiles.

For the uninfected group where all three blood sources could be compared, there was a significant effect of blood source on development time (F_(2, 16)_ = 19.46, *p* < 0.001). Larval development of the uninfected group held on human blood was consistently faster than that of the pig group and to a lesser extent the sheep group, regardless of sex (Figure 5A,B). There was no significant effect of sex (F_(1, 16)_ = 3.83, *p* = 0.068) or any interaction between sex and blood source (F_(2, 16)_ = 0.09, *p* = 0.912). For the *w*AlbB lines, there was no significant effect of blood source on development time overall (F_(1, 12)_ = 2.76, *p* = 0.123) (Figure 5A,B).

#### 3.2.2. *Wolbachia* Density (F1)

**Figure 6:**
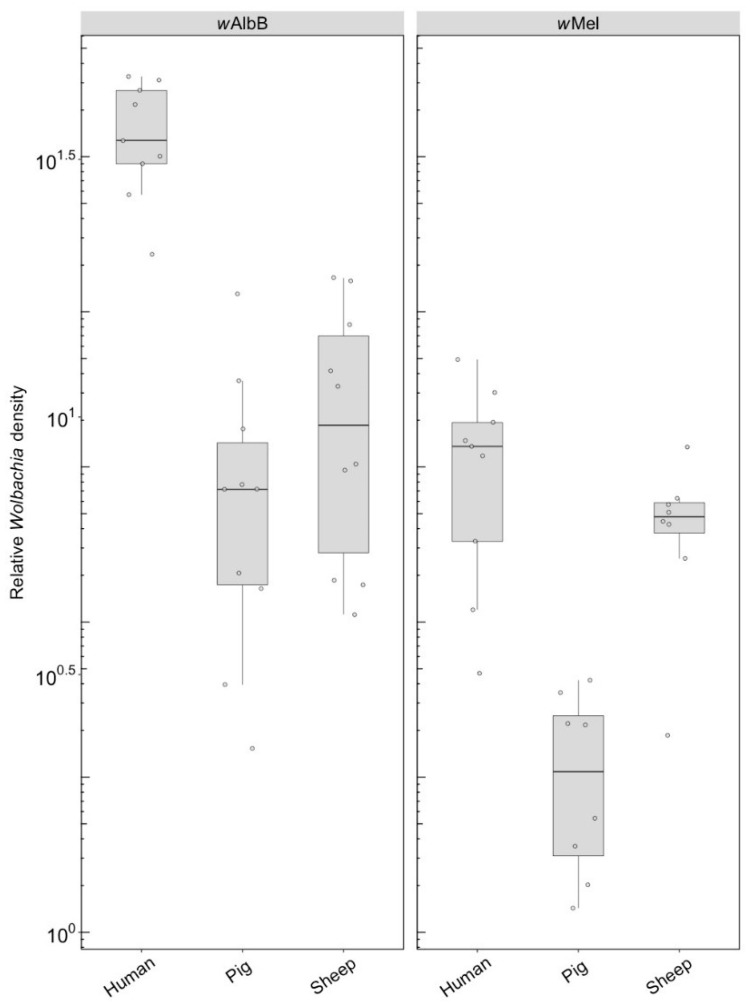
Comparison of the relative *Wolbachia* density of the infections types (*w*Mel, *w*AlbB) across different blood sources (human, pig, sheep) in the F1s that represent the offspring of parents exposed to their respective blood source. Each dot represents the density of an individual mosquito. The boxplots represent the first to the third quartiles around the median (horizontal grey line) and the vertical bars represent the 1.5 interquartile of the lower and upper quartiles. Between 8 and 10 F1s per blood source were tested.

F1s from parents fed on either pig or sheep blood showed a two to four-fold drop in density compared to parents that fed on this blood (c.f. Figure 6 with Figure 3). Differences in density were highly significant depending on blood source. For *w*AlbB, there was a highly significant difference (F_(2, 26)_ = 53.98, *p* < 0.001) with a 5-fold density difference between parents fed pig and human blood and a 3.5-fold difference between parents fed sheep and human blood. In contrast, exposure to different blood in parental mosquitoes resulted in similar densities of this infection across groups (Figure 3). For *w*Mel, there was also a significant difference among blood sources (F_(2, 24)_ = 19.08, *p* < 0.001). A decrease in F1s from parents with pig and sheep blood feeding had previously been evident from direct feeding for this infection type (Figure 3), although, in the F1s, the density on pig was lower than on sheep, whereas this was reversed under direct exposure.

#### 3.2.3. Fitness Effects (F10, After Feeding on Human Blood)

**Figure 7:**
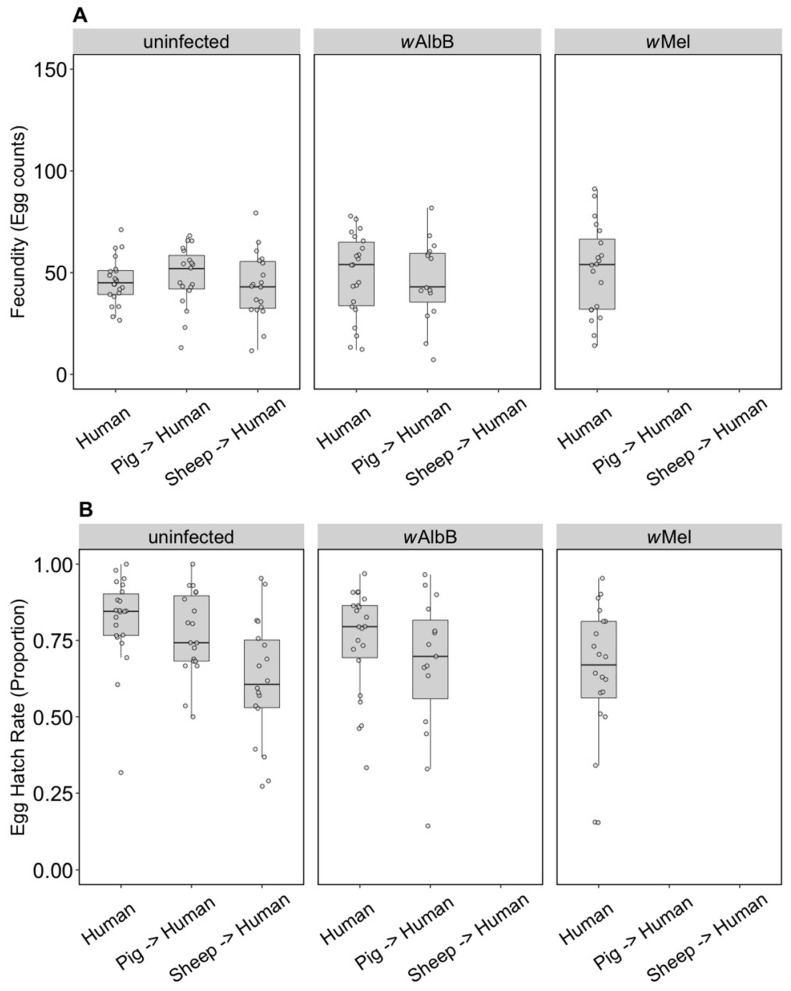
Comparison of fecundity and egg hatch rates across infection types (uninfected, *w*Mel, *w*AlbB) when females were fed on human blood after colonies were maintained on different blood sources for 10 generations (human, pig, sheep). Some of these groups lost their infection during maintenance; therefore, no comparison is possible for *w*AlbB and *w*Mel-infected groups fed on sheep blood. There is also no *w*Mel-infected group maintained on pig blood. (**A**) Fecundity (egg counts); (**B**) egg hatch rate (proportion). Each dot embodies the egg count and egg hatch rate of an individual female. The boxplots represent the first to the third quartiles around the median (horizontal grey line) and the vertical bars represent the 1.5 interquartile of the lower and upper quartiles. Around 15–24 females per blood source and generation were tested.

Some groups placed on the different blood sources were successfully maintained until generation 10, consistent with an ability of these populations to adapt to blood sources once they are established. We were then interested in testing whether there was any fitness cost when groups were switched back to human blood. For the uninfected line, there was no change in fecundity associated with blood feeding selection (F_(2, 57)_ = 0.52, *p* = 0.596) (Figure 7A), but there was an effect on egg hatch rate (F_(2, 56)_ = 7.16, *p* = 0.002) due only to sheep compared to the other two blood sources (Figure 7B) in a post hoc analysis. For the *w*AlbB infection type, there was no change in fecundity associated with blood feeding selection on pig versus human (F_(1, 35)_ = 0.15, *p* = 0.700) and egg hatch rate was also similar (F_(1, 35)_ = 1.43, *p* = 0.240) (Figure 7A,B).

#### 3.2.4. *Wolbachia* Density (F10, After Feeding on Human Blood)

**Figure 8:**
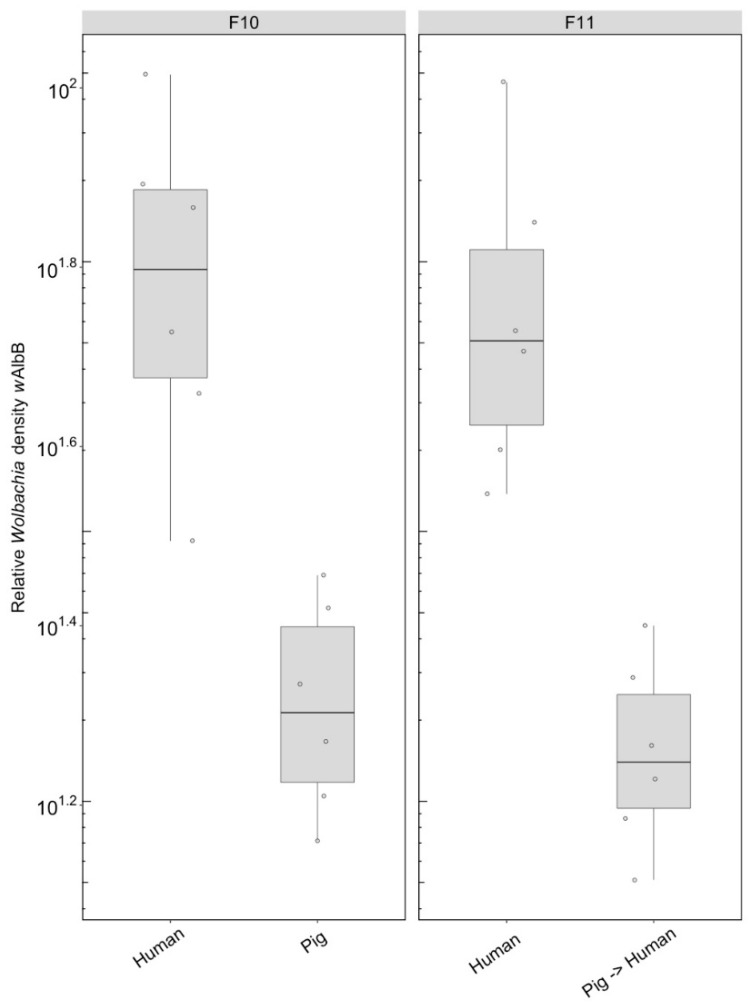
Comparison of the relative *Wolbachia* density of the *w*AlbB infections across the different blood sources (human, pig) after 10 generations of maintenance. Each dot represents the density of an individual mosquito. The boxplots represent the first to the third quartiles around the median (horizontal grey line) and the vertical bars represent the 1.5 interquartile of the lower and upper quartiles. Around six individuals per blood source were tested.

We compared the density of the *w*AlbB infection in mosquitoes fed on either human or pig blood for 10 generations as well as the effect on density after the line maintained on pig blood was re-introduced to human blood. There was a significant effect of the blood source on which the lines were maintained (F_(1, 10)_ = 15.93, *p* = 0.003), with density being lower when females were fed on pig blood (Figure 8). This difference in density was maintained after the pig blood-maintained group was fed on human blood in the following generation (F_(1, 10)_ = 15.65, *p* = 0.003) (Figure 8).

## 4. Discussion

This study indicates that key reproductive traits in *Ae. aegypti* can be lowered by a sheep or pig blood meal versus a human blood meal. While many mosquito species utilise a wide variety of blood sources to facilitate the egg laying process, *Ae. aegypti* is largely dependent on human blood for this purpose [24,34]. Mosquitoes fed on non-human blood showed a significant decrease in egg hatch rates compared to mosquitoes fed on human blood, regardless of their *Wolbachia* infection. Moreover, there appears to be a carry-over effect when the mosquitoes feed on human blood in the next generation. After reintroducing the F1 generation of each blood source (human, pig, sheep) to human blood, an increase in egg hatch rate was observed for the non-human parental exposure treatments. However, egg hatch rates of the group previously fed on sheep blood remained significantly lower than the groups exclusively fed on human blood. In addition to direct effects of the blood source on hatch rate, responses to non-human blood sources could also be affected by variation in blood volume ingestion between females fed on human and non-human blood sources, though this was not tested in this study. The use of a non-human blood source to maintain mosquitoes for release programs could not only lead to reduced fitness of the released mosquitoes, but may also reduce the reproductive success of the next generation.

Feeding on non-human blood sources also seems to limit the potential for optimal *Wolbachia*-infected *Ae. aegypti* colony maintenance, consistent with previous findings [24,35,36]. We were unable to sustain the *w*Mel groups on pig and sheep blood past the F3 and F2, respectively, due to low numbers of larvae. The *w*AlbB group maintained on sheep blood was also lost at the F3 stage. While larval population numbers of the *w*AlbB sheep group were high (two replicates, each with approximately 200 larvae), most of this group was found dead as 3rd–4th instar larvae. We are unclear about the reasons for this mortality in the groups, but perhaps *Wolbachia* generates additional costs on larvae when these are insufficiently provisioned after feeding on alternate blood sources. It is possible that in the absence of access to nutrients supplied by the blood meal, the *Wolbachia* infection may utilize key resources from the host. This is supported by the finding that *Ae. aegypti* cholesterol levels are reduced by 15–25% when carrying a *Wolbachia* infection compared to the absence of a *Wolbachia* infection [36]. In contrast, all the infected groups could be easily maintained on human blood for multiple generations. Other factors like inbreeding may also be involved if a low proportion of females in each population fed successfully across the generations, resulting in bottlenecks [28].

When considering long-term fitness effects, *Ae. aegypti* may experience if fed on non-human blood sources for several generations, we found a selection response in the uninfected groups where egg hatch rates on alternate blood sources were relatively high by F4. There was also an apparent increase in fecundity in the population held on pig blood, though egg hatch rates were still reduced. Moreover, the egg hatch rate effect persisted when mosquitoes were fed human blood again even after 10 generations of maintenance, suggesting a long-term fitness cost.

For the effects of blood meal on *Wolbachia* density, we found different patterns for the two infection types (*w*Mel & *w*AlbB) as well as differences between short- and long-term effects. After one generation on either pig or sheep blood, *w*Mel showed a significant drop in density, whereas the density of *w*AlbB remained similar. Both infection types showed no increase in density after being reintroduced to human blood. Considering the long-term effect on the *Wolbachia* infection, we found a 4-fold drop in density of *w*AlbB and *w*Mel when fed on either sheep or pig blood. Previous research demonstrates that endosymbionts often induce a reduction in host cholesterol levels [37,38]. This could be due to competition between the host (mosquito) and the *Wolbachia* endosymbiont for key nutrients supplied by the blood meal, yet there is also a high degree of disparity between the cholesterol and amino acids between human and other animal blood sources [36]. This could in part explain the reduction in *Wolbachia* density when mosquitoes are maintained on non-human blood. Caragata et al. [36] identified a large reduction in fecundity and hatch rate of eggs produced by *w*MelPop and *w*Mel-infected *Ae. aegypti* following a non-human (rat) blood meal. When the *Wolbachia*-infected *Ae. aegypti* colonies were fed on rat blood but supplemented with additional amino acids, fecundity increased by approximately 15–20 eggs and egg hatch rate increased by 30–40%. This suggests that a rescue effect may be possible if adaptation to the alternative blood source cannot be achieved to reach the phenotypic output observed on human blood. Groups that are seeking to mass rear *Ae. aegypti* may still be able to produce locally competitive mosquitoes with non-human blood if a supplementation of key nutrients such as amino acids is supplied.

There are two main methods for controlling arbovirus transmission using *Wolbachia*-infected *Ae. aegypti* currently being implemented. The first method involves population suppression through the release of mass quantities of *Wolbachia*-infected males into uninfected populations. *Wolbachia*-infected males mate with uninfected females, rendering the offspring inviable due to cytoplasmic incompatibility [7,39,40]. We have not tested the effects of alternate blood sources on incompatibility in our experiments, though incompatibility associated with *w*AlbB remains strong when males come from colonies that have been maintained on other blood sources (Nazni, pers. comm.). The second method involves replacement of uninfected *Ae. aegypti* populations with *Wolbachia*-infected mosquitoes which then interfere with arboviral replication and transmission [41]. We found that *Wolbachia*-infected *Ae. aegypti* raised in the laboratory using non-human blood sources exhibit reduced *Wolbachia* density and fitness relative to mosquitoes maintained on human blood. When mosquitoes maintained on non-human blood are released into field settings, the ability of *Wolbachia*-infected *Ae. aegypti* to replace naïve target populations may be diminished [42]. A decrease in *Wolbachia* density could also influence the ability of *Wolbachia* to block disease transmission, which seems to relate at least partly to *Wolbachia* density in the host [18]. Should invasion succeed, the ability of *Wolbachia*-infected *Ae. aegypti* to supress arboviral transmission may be reduced, but only if low *Wolbachia* densities are inherited.

## 5. Conclusions

*Aedes aegypti* fed on pig and sheep blood suffered from reduced fitness and *Wolbachia* density. Laboratory populations could adapt to feeding on alternative blood sources but in some cases this resulted in large bottlenecks. Deleterious effects on fitness and *Wolbachia* density persisted even after mosquitoes were returned to feeding on human blood. Maintaining *Ae. aegypti* on alternative blood sources in the short- or long-term will likely compromise their fitness when released into the field for disease control programs.

## References

[1] Attardo G.M., Hansen I.A., Raikhel A.S. (2005). Nutritional regulation of vitellogenesis in mosquitoes: Implications for anautogeny. Insect Biochem. Mol. Biol..

[2] Washino R., Tempelis C. (1983). Mosquito host bloodmeal identification: Methodology and data analysis. Annu. Rev. Entomol..

[3] Lyimo I.N., Ferguson H.M. (2009). Ecological and evolutionary determinants of host species choice in mosquito vectors. Trends Parasitol..

[4] Scott T.W., Takken W. (2012). Feeding strategies of anthropophilic mosquitoes result in increased risk of pathogen transmission. Trends Parasitol..

[5] Bhatt S., Gething P.W., Brady O.J., Messina J.P., Farlow A.W., Moyes C.L., Drake J.M., Brownstein J.S., Hoen A.G., Sankoh O. (2013). The global distribution and burden of dengue. Nature.

[6] WHO (2009). Dengue Guidelines for Diagnosis, Treatment, Prevention and Control: New Edition.

[7] Hoffmann A., Montgomery B., Popovici J., Iturbe-Ormaetxe I., Johnson P., Muzzi F., Greenfield M., Durkan M., Leong Y., Dong Y. (2011). Successful establishment of *Wolbachia* in *Aedes* populations to suppress dengue transmission. Nature.

[8] McGraw E.A., O’Neill S.L. (2013). Beyond insecticides: New thinking on an ancient problem. Nat. Rev. Microbiol..

[9] Ferguson N.M., Kien D.T.H., Clapham H., Aguas R., Trung V.T., Chau T.N.B., Popovici J., Ryan P.A., O’neill S.L., McGraw E.A. (2015). Modeling the impact on virus transmission of *Wolbachia*-mediated blocking of dengue virus infection of *Aedes aegypti*. Sci. Transl. Med..

[10] Dutra H.L.C., Rocha M.N., Dias F.B.S., Mansur S.B., Caragata E.P., Moreira L.A. (2016). *Wolbachia* blocks currently circulating Zika virus isolates in Brazilian *Aedes aegypti* mosquitoes. Cell Host Microbe.

[11] Moreira L.A., Iturbe-Ormaetxe I., Jeffery J.A., Lu G., Pyke A.T., Hedges L.M., Rocha B.C., Hall-Mendelin S., Day A., Riegler M. (2009). A *Wolbachia* symbiont in *Aedes aegypti* limits infection with dengue, Chikungunya, and Plasmodium. Cell.

[12] Bhattacharya T., Newton I.L., Hardy R.W. (2017). *Wolbachia* elevates host methyltransferase expression to block an RNA virus early during infection. PLoS Pathog..

[13] Caragata E.P., Dutra H.L.C., Moreira L.A. (2016). Inhibition of Zika virus by *Wolbachia* in *Aedes aegypti*. Microb. Cell.

[14] Geoghegan V., Stainton K., Rainey S.M., Ant T.H., Dowle A.A., Larson T., Hester S., Charles P.D., Thomas B., Sinkins S.P. (2017). Perturbed cholesterol and vesicular trafficking associated with dengue blocking in *Wolbachia*-infected *Aedes aegypti* cells. Nat. Commun..

[15] McMeniman C.J., Lane R.V., Cass B.N., Fong A.W., Sidhu M., Wang Y.-F., O’neill S.L. (2009). Stable introduction of a life-shortening *Wolbachia* infection into the mosquito *Aedes aegypti*. Science.

[16] Walker T., Johnson P., Moreira L., Iturbe-Ormaetxe I., Frentiu F., McMeniman C., Leong Y., Dong Y., Axford J., Kriesner P. (2011). The *w*Mel *Wolbachia* strain blocks dengue and invades caged *Aedes aegypti* populations. Nature.

[17] Xi Z., Khoo C.C., Dobson S.L. (2005). *Wolbachia* establishment and invasion in an *Aedes aegypti* laboratory population. Science.

[18] Ant T.H., Herd C.S., Geoghegan V., Hoffmann A.A., Sinkins S.P. (2018). The *Wolbachia* strain *w*Au provides highly efficient virus transmission blocking in *Aedes aegypti*. PLoS Pathog..

[19] Caspari E., Watson G. (1959). On the evolutionary importance of cytoplasmic sterility in mosquitoes. Evolution.

[20] Dutra H.L.C., Rodrigues S.L., Mansur S.B., de Oliveira S.P., Caragata E.P., Moreira L.A. (2017). Development and physiological effects of an artificial diet for *Wolbachia*-infected *Aedes aegypti*. Sci. Rep..

[21] Gonzales K.K., Hansen I.A. (2016). Artificial diets for mosquitoes. Int. J. Environ. Res. Public Health.

[22] Gonzales K.K., Rodriguez S.D., Chung H.N., Kowalski M., Vulcan J., Moore E.L., Li Y.Y., Willette S.M., Kandel Y., Van Voorhies W.A. (2018). The Effect of SkitoSnack, an Artificial Blood Meal Replacement, on *Aedes aegypti* Life History Traits and Gut Microbiota. Sci. Rep..

[23] Baughman T., Peterson C., Ortega C., Preston S.R., Paton C., Williams J., Guy A., Omodei G., Johnson B., Williams H. (2017). A highly stable blood meal alternative for rearing *Aedes* and *Anopheles* mosquitoes. PLoS Negl. Trop. Dis..

[24] Suh E., Fu Y., Mercer D.R., Dobson S.L. (2016). Interaction of *Wolbachia* and bloodmeal type in artificially infected *Aedes albopictus* (Diptera: Culicidae). J. Med. Entomol..

[25] McMeniman C.J., Hughes G.L., O’Neill S.L. (2011). A *Wolbachia* symbiont in *Aedes aegypti* disrupts mosquito egg development to a greater extent when mosquitoes feed on nonhuman versus human blood. J. Med. Entomol..

[26] Axford J.K., Ross P.A., Yeap H.L., Callahan A.G., Hoffmann A.A. (2016). Fitness of *w*AlbB *Wolbachia* infection in *Aedes aegypti*: Parameter estimates in an outcrossed background and potential for population invasion. Am. J. Trop. Med. Hyg..

[27] Ross P.A., Axford J.K., Richardson K.M., Endersby-Harshman N.M., Hoffmann A.A. (2017). Maintaining *Aedes aegypti* Mosquitoes Infected with *Wolbachia*. J. Vis. Exp..

[28] Ross P.A., Endersby-Harshman N.M., Hoffmann A.A. (2017). A comprehensive assessment of inbreeding and laboratory adaptation in *Aedes aegypti* mosquitoes. bioRxiv.

[29] Luo Y.P. (2014). A novel multiple membrane blood-feeding system for investigating and maintaining *Aedes aegypti* and *Aedes albopictus* mosquitoes. J. Vector Ecol..

[30] Lee S.F., White V.L., Weeks A.R., Hoffmann A.A., Endersby N.M. (2012). High-throughput PCR assays to monitor *Wolbachia* infection in the dengue mosquito (*Aedes aegypti*) and *Drosophila simulans*. Appl. Environ. Microbiol..

[31] Yeap H., Rašić G., Endersby-Harshman N., Lee S., Arguni E., Le Nguyen H., Hoffmann A. (2016). Mitochondrial DNA variants help monitor the dynamics of *Wolbachia* invasion into host populations. Heredity.

[32] Richardson K.M., Griffin P.C., Lee S.F., Ross P.A., Endersby-Harshman N.M., Schiffer M., Hoffmann A.A. (2018). A *Wolbachia* infection from *Drosophila* that causes cytoplasmic incompatibility despite low prevalence and densities in males. Heredity.

[33] R Development Core Team (2016). R: A Language and Environment for Statistical Computing.

[34] Scott T.W., Chow E., Strickman D., Kittayapong P., Wirtz R.A., Lorenz L.H., Edman J.D. (1993). Blood-feeding patterns of *Aedes aegypti* (Diptera: Culicidae) collected in a rural Thai village. J. Med. Entomol..

[35] Moreira L.A., Yixin H.Y., Turner K., Eyles D.W., McGraw E.A., O’Neill S.L. (2011). The *w*MelPop strain of *Wolbachia* interferes with dopamine levels in *Aedes aegypti*. Parasit Vectors.

[36] Caragata E.P., Rancès E., O’Neill S.L., McGraw E.A. (2014). Competition for amino acids between *Wolbachia* and the mosquito host, *Aedes aegypti*. Microb. Ecol..

[37] Lin M., Rikihisa Y. (2003). *Ehrlichia chaffeensis* and *Anaplasma phagocytophilum* lack genes for lipid a biosynthesis and incorporate cholesterol for their survival. Infect. Immun..

[38] Watarai M., Makino S.-I., Michikawa M., Yanagisawa K., Murakami S., Shirahata T. (2002). Macrophage plasma membrane cholesterol contributes to *Brucella abortus* infection of mice. Infect. Immun..

[39] Berman R. Scientists Introduce 20 Million Sterile Mosquitoes into California. http://bigthink.com/robby-berman/scientists-introduce-20-million-sterile-mosquitoes-into-california.

[40] Zhang D., Lees R.S., Xi Z., Bourtzis K., Gilles J.R. (2016). Combining the Sterile Insect Technique with the Incompatible Insect Technique: III-robust mating competitiveness of irradiated triple *Wolbachia*-infected *Aedes albopictus* males under semi-field conditions. PLoS ONE.

[41] Jeffries C.L., Walker T. (2016). *Wolbachia* biocontrol strategies for arboviral diseases and the potential influence of resident *Wolbachia* strains in mosquitoes. Curr. Trop. Med. Rep..

[42] Joubert D.A., Walker T., Carrington L.B., De Bruyne J.T., Kien D.H.T., Hoang N.L.T., Chau N.V.V., Iturbe-Ormaetxe I., Simmons C.P., O’Neill S.L. (2016). Establishment of a *Wolbachia* superinfection in *Aedes aegypti* mosquitoes as a potential approach for future resistance management. PLoS Pathog..

